# Feasibility of an online platform delivery of pulmonary rehabilitation for individuals with chronic respiratory disease

**DOI:** 10.1136/bmjresp-2021-000880

**Published:** 2021-03-24

**Authors:** Adam Lewis, Ellena Knight, Matthew Bland, Jack Middleton, Esther Mitchell, Kate McCrum, Joy Conway, Elaine Bevan-Smith

**Affiliations:** 1 Health Sciences, Brunel University London, London, UK; 2 Gloucestershire Health and Care NHS Foundation Trust, Brockworth, UK; 3 Brunel University London, Uxbridge, UK; 4 University of Gloucestershire, Cheltenham, UK; 5 Centre for Health, Medicine and Life Sciences, Brunel University London, Uxbridge, UK

**Keywords:** pulmonary rehabilitation, COVID-19, emphysema

## Abstract

**Introduction:**

SARS-CoV-2 has restricted access to face-to-face delivery of pulmonary rehabilitation (PR). Evidence suggests that telehealth-PR is non-inferior to outpatient PR. However, it is unknown whether patients who have been referred to face-to-face programmes can feasibly complete an online-PR programme.

**Methods:**

This service evaluation used a mixed-methods approach to investigate a rapid PR service remodelling using the University of Gloucestershire eLearn Moodle platform. Quantitative baseline demographic and PR outcome data were collected from online-PR participants, and semistructured interviews were completed with PR staff and participants.

**Results:**

Twenty-five individuals were eligible from a PR waiting list. Thirteen declined participation and 14 completed PR. Significant pre-post online PR improvements were achieved in 1 min sit-to-stand (CI 2.1 to 9 (p=0.004)), Generalised Anxiety Disorder (CI −0.3 to −2.6 (p=0.023)), Primary Health Questionnaire-9 (CI −0.3 to −5.1 (p=0.029)), Chronic Respiratory Questionnaire dyspnoea (CI 0.5 to 1.3 (p=0.001)), fatigue (CI 0.7 to 2 (p=0.0004)), emotion (CI 0.7 to 1.7 (p=0.0002)), mastery (CI 0.4 to 1.3 (p=0.001)). Interviews indicated that patient PR inclusion was made possible with digital support and a PR introduction session improved participant engagement and safety. Incremental progression of exercise was perceived as more successful online compared with face-to-face PR. However, perceptions were that education sessions were less successful. Online-PR required significant staff time resource.

**Discussion:**

Online-PR improves patient outcomes and is feasible and acceptable for individuals referred for face-to-face PR in the context of a requirement for social distancing. Face-to-face programmes can be adapted in a rapid fashion with both staff and participants perceiving benefit. Future pragmatic trials are now warranted comparing online-PR including remote assessments to centre-based PR with suitably matched outcomes, and patient and staff perceptions sought regarding barriers and facilitators of online delivery.

**Key messages**

- Can patients on pulmonary rehabilitation (PR) waiting lists feasibly complete online-PR programmes? If so, how do staff normalise the process of providing online-PR within existing NHS services?
- Online-PR is deliverable, patients think it is feasible and patient outcomes are improved.
- To our knowledge, this is the first online-PR evaluation using staff experiences, feedback from patients and PR outcome data, using a novel online platform not previously used in the context of PR.

## Background

During the SARS-CoV-2 pandemic, face-to-face delivery of pulmonary rehabilitation (PR) stopped due to UK national lockdown and social distancing rules. People with chronic respiratory disease (CRD) have suffered as a result, particularly psychological impacts including anxiety, loneliness and concerns about personal health.^1^ Loneliness, domestic isolation and social disengagement are longitudinally associated with poorer physical performance in older adults^2^ and shielding during COVID-19 has reduced physical activity levels of patients with CRD.^3^ Therefore, it is important to enable individuals to continue receiving interventions which promote physical activity, which are useable for staff and patients during the pandemic. Evidence suggests that providing home PR is feasible and comparably effective to face-to-face delivery when performed as part of a randomised controlled trial (RCT).^4–7^ Furthermore, tele-rehabilitation has previously been shown to improve exercise capacity, symptoms and psychological comorbidity in patients with COPD as part of an RCT.^8^ It has also been shown that other group programmes for individuals with CRD can be feasibly delivered online such as Singing for Lung Health groups.^9^ The Association of Chartered Physiotherapists in Respiratory Care state that only 50% of PR programmes surveyed provide remote-PR via video-conferencing or web-based platforms and report that it is essential that such options are evaluated following implementation.^10^ Other national survey data suggest that only 22% of clinicians surveyed provide remote-PR.^11^ The two web-based platforms currently recommended for remote-PR include myCOPD and SPACE for COPD.^10^ Further platforms warrant evaluation. We aim to provide such an evaluation, focused on programme outcomes, staff normalisation of online-PR delivery within other service demands, and we sought patient feedback regarding feasibility.

## Method

The study design used a mixed-methods approach. Qualitative data were analysed using thematic analysis of patient and staff experiences.^12^ Participants provided consent to participate. The deductive analysis of staff experiences was specifically aligned to normalisation process theory (NPT).^13^ According to May and Finch^13^ NPT ‘is concerned with the social organization of the work (implementation), of making practices routine elements of everyday life (embedding), and of sustaining embedded practices in their social contexts (integration)’. The social context within this study related to the organisation and practices of a Community Respiratory Team. Interview questions were aligned to areas of intervention content and delivery, design conduct and process and outcomes.^14^ Data were familiarised, listening to the interviews repeatedly, writing, reading and re-reading transcripts. The transcripts were then tagged with phrases using the comment function in MS word. Codes were then transferred and grouped into larger meaning units which were then reviewed once all transcripts had been coded. Themes were developed, reviewed and defined on review of the codes in relation to the reference text from the interview and understanding of NPT. Semistructured interview guides are provided in the online supplemental appendices. Staff are referred to by participant number and online-PR participants have been given pseudonyms.

10.1136/bmjresp-2021-000880.supp1 Supplementary data

### Statistical analysis

Quantitative analyses were performed using SPSS 26. Feasibility outcomes of attendance were calculated with percentages. Feasibility was determined according to UK National PR Audit data whereby 42% of those referred to a programme completed PR.^15^ Normality of other outcome data was assessed visually according to histogram and box plots in combination with assessment of the Shapiro-Wilk test at a significance level of p<0.05. Accordingly, independent sample t-tests, Mann Whitney U and χ² tests were performed to compare demographics of those who were assessed compared with those who declined participation on the online-PR. Paired sample t-tests and Wilcoxon signed rank tests were performed for baseline and follow-up objective outcome measures.

### Patient and public involvement

Patients and members of the public were not involved in the development of this project due to the rapid remodelling of service delivery. However, staff and participant views contained within this service evaluation will help inform further research.

### Procedures

The learning management system used was Moodle, named ‘eLearn’ within the University of Gloucestershire. There was functionality for video conferencing, messaging groups and individuals with text using the keyboard and a messaging pane, or via microphones embedded within the computer. Further information about the online platform is provided in the online supplemental appendices. Assessments were carried out virtually at a time convenient to the participant. A full history of present condition, medical history, drug and social history, a detailed falls history and falls checklist was completed. Inhaler technique was checked, pulse oximetry and all outcome measures were taken during these assessments. The online-PR exercises were developed by an exercise specialist and groups were moderated by clinicians. Further details of the exercise component and risk assessment are provided in the online supplemental appendices. The online course was provided to patients two times a week for 6 weeks and patients also received one-to-one phone calls with a clinician at weeks two and four. Further details of the online programme are found in table 1.

**Table 1:** Adaptions made for online delivery of PR

Traditional face to face programme	Online-PR delivery
Face-to-face patient assessments	Online video-based assessments
Incremental shuttle walk test exercise capacity outcome	1 min sit-to-stand exercise capacity outcome
Progression to 3 min per endurance exercise	Progression to 4 min per endurance exercise
Clinician led exercise	Exercise instructor led exercise
Resistance exercises with free weights	Resistance exercises with Theraband
Group education delivery within sessions	Separate individually accessed education
No preliminary patient home visit	Patient home visit for equipment delivery and IT platform training as needed
No prior equipment provided	Theraband, oximeter and sometimes IPad delivered
Home exercise programme administered on session one (paper based)	Home exercise programme administered once patient confident with online participation (paper based)
Community hall venues	Patient home venue
MDT education including: Understanding your lung condition, breathlessness management including input from psychological therapist, cough and sputum, planning for future, nutrition, benefits of exercise, hospital care, medications and inhaler technique	MDT education including: Understanding your lung condition, breathlessness management including input from psychological therapist, cough and sputum, planning for future, nutrition, benefits of exercise, hospital care, medications and inhaler technique.
Introduction session before preassessment including expert patient experience	Introduction session following pre-assessment led by exercise specialist and clinician
Paper based Patient-rReported Outcome Measures, missing data entry possible	Digitally completed outcome measures, submission not possible without complete data entry.
Clinical notes written on System one	Clinical notes written on System one

PR, pulmonary rehabilitation.

### Participants

Participants with CRD who were referred to the community respiratory team for face-to- face PR were screened for eligibility to participate according to British Thoracic Society guidance.^16^ Eligible potential participants were recruited from caseloads of cancelled PR classes and invited to attend by telephone. Convenience sampling was used for interviews of participants with CRD and staff members of the online-PR programme.

## Results

Thirty patients were screened and 25 fit the eligibility criteria for PR. Thirteen patients declined commencing online-PR (no internet access (n=3), low confidence in using technology (n=3), personal preferences (n=3), four of whom had undocumented reasons, two felt self-conscious using web-cameras). Seventeen were assessed and started the programme and 14 patients completed at least 9 out of 12 sessions and therefore deemed completers. There were no adverse events. Clinicians moderating groups were able to take participants into a breakout space if an adverse event were to occur. The moderator had access to participant and next of kin contact details. Further details are provided in the participant flow diagram (online supplemental appendices figure 1).

### Baseline demographics

Table 2 presents the baseline demographics of participants.

**Table 2:** Participant baseline demographics

Demographics	Started online-PR (n=17), Mean (SD)/median (IQR)	Declined online-PR (n=13), Mean (SD)/ median (IQR)
Gender ♀♂	9/8	6/7
Age	69.7 (10.7)	72.9 (10.8)
BMI	26.6 (13.6)	26.6 (10.4)
Diagnosis	15 COPD 1 ILD 1 Asthma	11 COPD 1 ILD 1 Asthma
MRC	3 (1)	3 (0.75)
Owned own computer/laptop	12 (70.5%)	7 (53.8%)
Previous face-to-face sessions	5 (4.*5*)	5 (5.5)

BMI, body mass index; MRC, Medical Research Council.

### Quantitative outcomes

Table 3 presents pre-post PR outcome data.

**Table 3:** Outcome measure changes from participating in online PR

	Baseline (n=14)	6-week follow-up (n=14)	Delta	CI (p value)
1 min STS	15.5 (5.3)	21.1 (7.8)	5.6 (6)	2.1 to 9 (0.004)
GAD	4.8 (4.6)	2.7 (3.3)	−2.1 (3)	−0.3 to −2.6 (0.023)
PHQ	7.9 (5.1)	5.2 (5.5)	−2.7 (4.1)	-0.3 to -5.1 (0.029)
CRQ dyspnoea	3 (0.9)	3.9 (1.1)	0.9 (0.7)	0.5 to 1.3 (0.001)
CRQ fatigue	3.3 (1)	4.7 (1.3)	1.4 (1.1)	0.7 to 2 (0.0004)
CRQ emotion	4 (1)	5.2 (0.9)	1.2 (0.9)	0.7 to 1.7 (0.0002)
CRQ mastery	4.4 (1.1)	5.3 (1)	0.9 (1.3)	0.4 to 1.3 (0.001)

CRQ, chronic respiratory disease; GAD, generalised anxiety disorder; 1 min STS, One min sit to stand; PHQ, Primary Health Questionnaire; PR, pulmonary rehabilitation.

These data indicate that 6 weeks of online-PR participation significantly improved all outcome measures of exercise capacity, anxiety, depression and respiratory related quality of life.

### Qualitative data—staff

All four staff members providing online-PR were interviewed. Staff members included a team lead physiotherapist, other physiotherapist, nurse and exercise instructor. Analytic themes were aligned to the components of NPT including coherence, cognitive participation, collective action and reflexive monitoring.

#### Coherence (the meaningful qualities of practice)

On the background of patient deterioration, in the absence of other care provision, the ethos of providing online-PR was for it to be as inclusive as possible for patients. This inclusivity was made possible by significant digital optimisation, repeated communication between patients and staff, and continuity of care provided by team members.

If someone needed an iPad, we can’t obviously post that, I would have to go and show them how it all works and explain to them, get them logged in…that would take anywhere from half-an-hour to an hour at their house. (Participant 4)

The delivery of online-PR should be flexible for clinical workloads and alternate service provision while fitting into the daily lives of patients with respiratory disease.

We think it offers us the option to work slightly longer days or more flexibly…we think it’s an option for those people who potentially still in work who can’t come to a face-to-face group. (Participant 1)

Non-clinical staff engagement, commitment and leadership are essential, and patient safety and exercise progression as a group are of paramount concern.

It was unknown, we were taking a risk, so we kind of discussed a lot about safety, about keeping the patients safe, about having a risk assessment of the actual process. (Participant 3)

#### Cognitive participation (enrolment and engagement of individuals or groups)

Delivering face-to-face PR was not an option or feasible in relation to community spaces and patient appetite.

But I don’t think the risk appetite to do that will be there, in the real world actually, are we going to get five people who want to come to a group of people who cough and sneeze and splutter? (Participant 2)

Social distancing, we couldn’t replicate what we are delivering now in any kind of physical environment with restrictions. (Participant 1)

Plans for PR development were long-standing prior to the pandemic due to a lack of uptake in the traditional format. Patients were already becoming accustomed to using alternative digital platforms for other social affairs, although some frustration remained using IT. An iteratively designed introduction session improved engagement and safety.

Now the introductory session goes over you know very clearly what the expectations are if you’re an oxygen user. (Participant 1)

Adding in that introduction session definitely helped, as we were able to see who was having the tech issues and things beforehand. (Participant 4)

Unlike face-to-face PR, patients exercised as a group, which made delivery and monitoring easier, and improved overall volumes of exercise completed. Because of the high standard of work provided by non-clinical staff, once participants were set up on a programme there was limited clinical work involved, which enabled other services to benefit such as oxygen therapy prescription.

I don’t feel like I'm doing a huge amount of clinical work with this online stuff. (Participant 2)

However, a significant amount of staff time was required to enable the beneficial outcomes.

Once people are on the course, that’s kind of the easy part, I think its selecting people to get on, getting them to agree, then they’ve got to have a pre-assessment, and before they have a pre-assessment they’ll need their pulse oximeter and, after their pre-assessment they’ve got to be posted all the paperwork and things, we need to make sure everyone knows how to log onto eLearn…before they actually start is the most time consuming part. (Participant 4)

Now you’ve got to have a third person for the first, I don’t know four sessions to deal with the IT. (Participant 2)

#### Collective action (interaction with already existing practices)

Necessary adaptions to the service were required to cater for the frail, new oxygen users and those with IT issues, although all could be reasonably catered for.

They had this sense of achievement that they'd mastered technology…I saw more frustration with NHS transport getting patients to pulmonary rehab face-to-face than I have ever seen on you know online. (Participant 4)

Incremental progression of exercise was perhaps more successful than during face-to-face delivery, and levels of effort regarding breathlessness and perceived exertion were effectively monitored, with Borg scales incorporated into online delivery.

They were the same exercises each week, but we started with two minutes per exercise and over the course of the six weeks we increased it to four minutes so doubling their time. (Participant 4)

Patient outcomes improved accordingly. Exercise delivery was straight-forward. However, engagement in education was not as successful.

In a (face-to-face) group setting you’ll maybe recap week-to-week… ask them questions about what they’ve learnt before and judge their understanding, you don’t get to do that in the online world, the reality is although you phone them twice, you'll often say, “right what do you want to ask me, from what you’ve watched online” and they, 9/10 they’ll say “oh nothing”, so you’re not sure how much they're engaging in the education. (Participant 1)

If we can make the education a bit more bitesize, a bit more segmental, it might be beneficial so people can come back to it. (Participant 2)

They only need to click on the section for it to go green, they don’t have to watch the video. (Participant 4)

Expanded provision of online-PR was planned regarding winter pressures. However, it was not clear whether resource or other service demands would allow this.

#### Reflexive monitoring (how a practice is understood or assessed by actors in it)

Although outcomes were positive, not all outcomes were assessed, and the quality of care provided to patients compared with face-to-face PR should be questioned further. Prior to the offer of online-PR, patients were deteriorating and desperate for some provision of clinical support and with other parts of life locked down any offer was hugely well received. However, rapport between patients and staff was difficult to foster, holistic patient assessment was harder, cameras had to be muted and sessions were unidimensional regarding exercise.

Rapport you would normally have with a patient, I think you lose. (Participant 3)

If someone comes in and they’re very wheezy and they're struggling, I think they can hide it a bit more on the camera maybe and I don’t get to know the patients, so I can watch them exercise but I don’t get that engagement through delivering the education and what some of their other problems might be. I think one of the best things about doing the face-to-face PR with a clinician…is that we pick up on lots of little things that can improve someone’s condition whether that might be some different techniques they might want to try, changes to medication, other health things, signposting to different services things like that which I'm not sure we’ll pick up with an online course. (Participant 2)

Education engagement, delivery and assessment require significant improvement and innovation.

I think the education we can think much more carefully about…I think there’s scope to be really really creative, with the online platforms. I think we can look at sort of education theory that we would look at for university students, we could look at how do people learn. (Participant 3)

Individual patient attention was stifled at times because of technology, and patients were reluctant to engage with each other without clinician attendance. These potential pitfalls were put into context of an appetite to use what has been learnt from the online-PR service and continue to implement and adapt face-to-face delivery:

We don’t have the time to kind of ask patients about too much “how much did you access?, what bits did you enjoy?”…maybe I should delve a little bit into which bits they’ve engaged in or not to see how much they're engaging in it. (Participant 2)

The important ethos of the staff was continuing to offer a choice of participation for all:

Lots of people have questioned, “well what about people who haven’t got access to technology?” Which obviously makes it, if people haven’t got access, it does make it inequitable. However, I would come back at those people and say, “yeah, but in face-to-face you’ve also got the people who can’t get there, who are severely disabled” so by default traditional face-to-face pulmonary rehab could be deemed inequitable or if people are frightened, or lack confidence with groups of people. (Participant 3)

We feel what we are doing is the most sensible way for us to behave over winter. (Participant 1)

### Online-PR participant data

Four participants who completed online-PR delivery were interviewed. Two men and two women with an average age of 62 (SD: 13) all had COPD. Thematic analysis from these interviews developed three themes including digital literacy, effectiveness of programme and comparability of models. Further example quotes and codes are provided in the online supplemental appendices.

### Digital literacy

Any problems participants had with the technology could be overcome, and although frustrating, they were not perceived as insurmountable barriers.

I don’t find the tech that easy but once it’s up and running its OK.

(Neil)

Pictures on the site make it easier.

(Sheila)

Teething problems were also reported which reflects the staff experiences:

The first week wouldn’t work on laptop

(Neil)

### Effectiveness of programme

Patients also perceived the online programme to be beneficial, noticing functional improvements in their activities of daily living.

It encouraged me to get walking again … I started off with half a mile and the last one I did was 1.2 miles. I’m pleased with that, my goal is 2 miles.

(Neil)

I used to have a mattress downstairs and I don’t use it anymore. I do the housework now and garden. Huge difference.

(Rob)

Patients reported that there was ‘no choice’ and that they either participated in the online PR offer or received nothing.

### Comparability of models

Individuals found the online group comparable to face-to-face groups, stated some benefits of doing the exercises at home compared with in a group, but noted that group interaction was lacking.

There was no difference between doing it online or in a group.

(Sheila)

There are a few differences with the exercises but I found it (online) better. I was doing too much (exercise) in a group because it was longer. They (exercises) were the same time but we got more rest periods online.

(Rob)

I felt more comfortable at home doing the programme.

(Rob)

It would be better face-to-face but you’ve got to go with what’s available, a lot of it is outside of our control.

(Jackie)

## Discussion

This service evaluation indicates that providing online-PR for patients with CRD improves patient outcomes and is a feasible alternative to face-to-face delivery in the context of a requirement for social distancing. Seventeen out of 25 (68%) patients were able to transfer appropriately from face-to-face to online delivery during COVID-19 and 14/25 (56%) completed PR. Fourteen out of 17 (82.3%) enrolled completed, which also achieves more than the threshold National PR audit recommendation C3 of 70% completion.^17^ Hansen *et al*^5^ previously have shown that completion rates of tele-rehabilitation can be higher than face-to-face models when judged by participants remaining in either the tele-rehabilitation group (49/67) or traditional PR (43/67) for the full intervention period. Furthermore, a recent Cochrane review on tele-rehabilitation in CRDs concluded from a meta-analysis of three studies that individuals were more likely to complete a minimum percentage of prescribed sessions during tele-rehabilitation compared with face-to-face PR (OR 5.36, 95% CI 3.12 to 9.21; 516 participants).^18^ The completion threshold in our study is likely higher than the pooled minimum percentage used in the Cochrane review. Our mixed-methods findings in this study offer support for these figures in the context of SARS-CoV-2; this may occur because of limited resources and patient appetite as described in our study. Benzo *et al*^19^ performed a feasibility study of an 8-week video-based physical activity and health-coaching intervention for individuals with COPD. Their study indicated that patients were highly adherent to the home programme with high levels of satisfaction. In comparison to Benzo *et al*’s study,^19^ the exercise frequency was lower and intensity higher in our study. Furthermore, exercises were performed live in a group with supervision and assessment by physiotherapists, therefore meeting the definition of PR. Our study also used different outcome measures and qualitative analyses which further develop understanding. For example, Participant 4’s experience above offers further potential insight regarding Benzo *et al*’s^19^ report of 100% completion of many activities such as ‘watched how-to videos’. It is possible the participants pressed a button to indicate they completed this component without actually watching the videos.

There were no statistically significant differences between the online-PR starters and those who declined regarding baseline demographics in our study. 16.7% fewer online-PR decliners had their own PC or laptop compared with online-PR starters. This difference was not statistically significant (χ² test p=0.494). This could be a type two error in relation to the small sample size in this study.

Staff were able to normalise the process of online provision within their wider clinical service. There were barriers and limitations which were highlighted, including issues with IT, education provision and capability to provide patient support and quality of patient care and self-management. Issues with IT access, competency and motivation for an online format were reported. This reflects a recent survey results by Polgar *et al*^20^ who state that out of 193 PR service users 31% had never used the internet and 29% had no interest in using a digital platform. This contrasts somewhat from findings by Seidman *et al*,^21^ who reported that out of 254 patients with CRD surveyed 70% regularly use a computer or tablet and 60% were willing to use tele-rehabilitation. Our service evaluation shows that although some patients did not want to participate in an online programme, other issues of IT could be overcome, by providing personalised equipment and one-to-one technical support in patient homes. Other options are highlighting wifi-hubs in the community and contacting digital champions in primary care services for example. In fact, online-PR delivery has been reported as a solution to enabling improved patient digital health skills, by incorporating such information in education sessions.^22^ Furthermore, a previous pilot of home-based online-PR suggests that such platforms are useable by participants and economically viable.^23^

Quantitative results indicated that the service evaluation programme was successful at improving functional exercise capacity, anxiety and depression (which was clinically significant at baseline) and multiple domains of disease-specific health-related quality of life. A previous threshold has been established by Puhan *et al*^24^ in which those individuals who have a 1 min sit to stand test of at least 19.5 have a lower mortality risk at 2 years. Participants within this service evaluation crossed this threshold, as well as the MCID of three repetitions.^25^ This improvement may have been possible due to the focus of regular incremental increases in endurance exercise time. All individuals completed both physical and questionnaire-based outcomes successfully, both at baseline and follow-up, indicating that traditional PR outcomes are feasible and have transferability to an online delivery format. Nevertheless, 56% patient completion is suboptimal. Further research is needed to improve uptake and completion.

### Strengths and limitations

This service evaluation reports results from using the Elearn platform in the context of PR using remote and video-based patient assessments. The use of eLearn and the working partnership between academic and NHS institution is important. Once someone in the University organisation opens the platform for a clinical service, an unlimited amount of patients can benefit from its use at no additional cost. In the context of scaling up delivery, this will be an important consideration for many services grappling with increased patient workloads over the winter and continuing SARS-CoV-2 working practices. Furthermore, remote assessments are the most practical and relevant format for patients participating in online-PR. Previous trials in tele-rehabilitation have still required patients to attend face-to-face clinical assessments before and after PR which may not be fit for purpose in the COVID-19 era.

These results are from one clinical service evaluation, using one online digital platform, with a small sample size, and therefore may have limited external validity. Furthermore, the majority of participants had already participated in some face-to-face PR sessions. Moreover, there was no control group in the study and it is not clear to what extent outcomes would have changed with usual care.

### Future research

Further pragmatic trials are required whereby patients are offered the choice of face-to-face compared with online-PR delivery. Patient choice has been considered in similar comparison of home-based versus outpatient-based PR successfully,^26^ but regular video-based intervention was not part of the home-based intervention in this cohort study. Interventions should be matched for principles of exercise training and education provision, but necessary alterations are required for online delivery, regarding space available, instruction and patient individualisation of care, which all need to be considered. Further research is also required to understand the best methods of providing digitally delivered patient education.

## Conclusion

This service evaluation investigated the outcomes, staff normalisation practices and feasibility of providing an online PR programme during SARS-CoV-2 pandemic. Online-PR improved clinical outcomes and was feasible to deliver. Patients found it acceptable, and clinicians adapted their workloads and normalised the online delivery as part of ongoing service provision. Future pragmatic trials are now warranted and focusing on improving online education delivery as part of PR is essential.

## Data Availability

Data are available on reasonable request.
