# Supplementary material for: Feasibility of an online platform delivery of pulmonary rehabilitation for individuals with chronic respiratory disease
Source: BMJ Open Respir Res. 2021 Mar 24;8(1):e000880. doi: 10.1136/bmjresp-2021-000880 (PMC7993314; doi:10.1136/bmjresp-2021-000880)
Supplement: Supplementary data [file bmjresp-2021-000880supp001.pdf]

Appendices

Figure 1: Flow diagram of recruitment and completion of online PR: Consort diagram

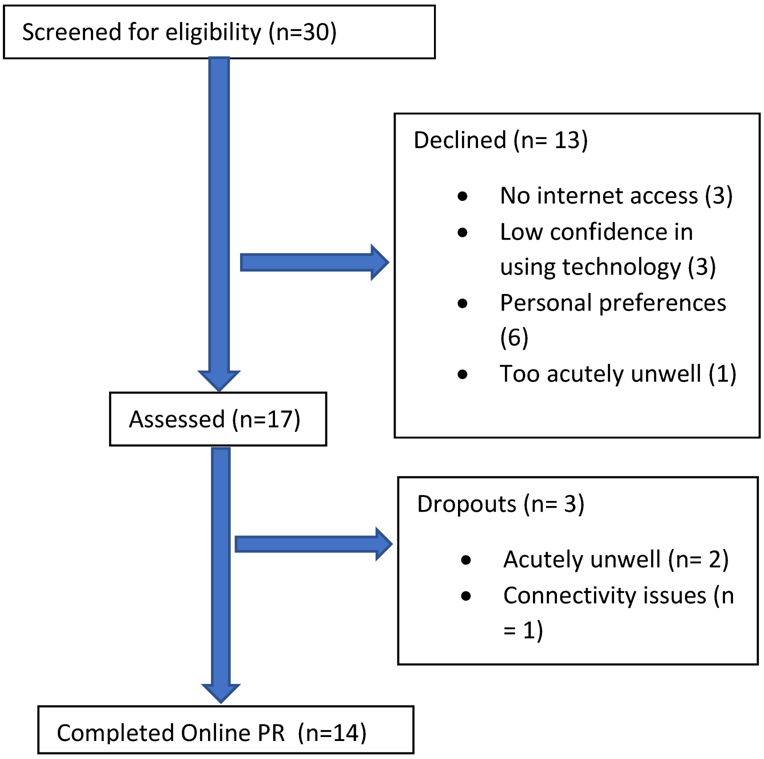

Online platform set-up and access

Remote delivery of the pulmonary rehabilitation programme was via an integrated system of learning platforms normally used for student education at the University of Gloucestershire (UOG). Access to the system for patients and pulmonary rehabilitation staff was gained via a username and password allocated by the university's Library Technology and Information (LTI) Service. The process was supported by a designated member of university staff (EBS) working alongside a Learning Technologist from the University Academic Development Unit (ADU). There were no additional cost implications for the university in terms of IT resources. Entry to the learning platform was via software called 'eLearn'; an externally facing version of Moodle ™. Moodle™ is an online learning platform, widely used in educational institutions, via which educators can create personalised learning environments for their students (https://moodle.com/about/). The platform is used creatively to provide a whole plethora of pedagogical interventions as it has the capacity to integrate other online systems once customised by the LTI service One of these systems is a web-conferencing platform called 'BigBlueButton' (https://bigbluebutton.org/) with its externally facing element called 'Greenlight'. Greenlight allows live-streaming of sessions to an audience and has familiar videoconferencing features such as chat / messaging, screenshare, digital whiteboard use, breakout rooms and screen presentations. The technological specifications of the digital platform and delivery process were scrutinised by the NHS IT department for safety and confidentiality in the context of data protection guidelines. Patients needed to have access to the internet via a suitably enabled digital device in order to participate. All the learning resources such as handouts, videos, links to internet resources etc were uploaded to eLearn. From the eLearn landing page patients would then click onto the Greenlight section which would open the livestreamed exercise and education sessions. Patients were able to switch device microphones on during each session for discussion.

Figure 2: Online PR delivery flow technical flow diagram

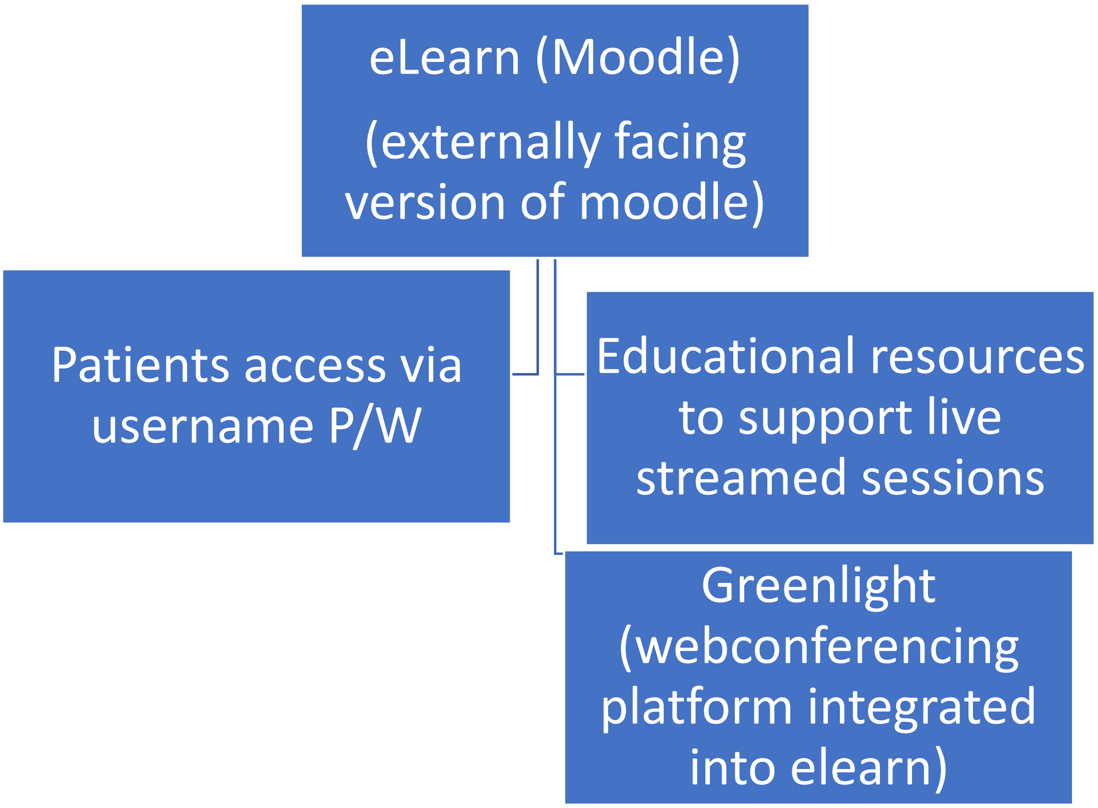

Staff Semi-structured interview guide

Consent.

Purpose of interview – experiences of set up, delivery and evaluation of online programme

1. Context – tell us a little about your current professional role and background in pulmonary rehabilitation

2. Tell me about your experience of setting up the programme for online delivery

   - What was difficult, what have you learnt, were there any surprises

3. What patients have been eligible? – currently enrolled, on waiting list, exacerbators

4. Talk me through what you have been doing regarding patient assessments and re-assessments.

   - What has gone well? Any concerns

5. Discuss the particular components that you have included as part of the programme
   - Exercise, education, home practice, other support.
   - What changes have been required compared to the traditional model

6. How does your practice in delivering the Pulmonary Rehabilitation compare with that of face-to-face delivery?
   - Working in an MDT?
   - Working with patients
   - Governance and organisation?
   - Thoughts on evidence based practice?

7. What onward support or advice do you give patients at the end of the programme

Online-PR participant semi-structured interview

General into

How are you feeling today?

Tell me a little about yourself

Online- PR

Before the COVID crisis did you attend any group therapy sessions for your condition for example Pulmonary rehabilitation / singing groups / exercise classes?

What are your thoughts about doing a group class online?

Talk to me about what you think the differences are?

Is there anything you've not really thought of?

Where I think something is useful, can follow this thread

How you felt it went

What benefits if any are you getting?

What do you mean by this?

Tell me more

Can you give examples?

Technology

Discuss ease of technology

How have you found the technology?

Zoom classes and Elearn access

Have you used this sort of technology before?

Format of the programme

Talk to me about the format of the rehab

Is there anything really good?

Is there anything that can go?

Online-PR participant example coding and quotes

Themes	Codes	Supporting statements
Digital literacy	Teething problems with required technology for PR programme<br><br>Relationship between participant's digital literacy and digital competence<br><br>Facilitation from staff and family<br><br>Elicited success<br><br>Simplicity of the online platform facilitates participant use	*" the first week wouldn't work on laptop".*<br><br>*"I don't find the tech that easy but once it's up and running its OK"*<br><br>*"Pictures on the site make it easier".*<br><br>*"Got it set up on my son's laptop then it was working OK – my son's more technically minded than me".*<br><br>*"I had problems logging on -II tend to do it on my son's laptop".*
Effectiveness of programme	All the components of the programme effective<br><br>Exercise<br><br>Education<br><br>psychological interventions)<br><br>perceived as beneficial.<br><br>Perceived improvements in traditional PR outcomes<br><br>Increased levels of physical activity<br><br>Breathlessness<br><br>mental health<br><br>confidence and motivation	*"It encouraged me to get walking again … I started off with half a mile and the last one I did was 1.2 miles. I'm pleased with that my goal is 2 miles".*<br><br>*"I've benefitted because I can shower, walk around house, take the dog for short walk".*<br><br>*"It's really good. It's helped my mental health. I'm not so depressed".*<br><br>*"I've more motivation to move about".*<br><br>*"I used to have mattress downstairs and I don't use it anymore. I do the housework now and garden. Huge difference".*<br><br>*"These courses are amazing. They give you more information than the doctors. I learnt things I didn't know - my BMI for example".*<br><br>*"It's amazing – anyone with my condition - I wish they would do this course; it motivates you".*
Comparability of models	Online PR comparable to face to face PR.<br><br>Favourable experience of online PR in context of global pandemic.<br><br>Individual exercise progression<br><br>Group interaction occurs online but less so than face to face PR	*"There was no difference between going it (PR) online or in a group"*<br><br>*"There are a few differences with the exercises but I found it (online) better. I was doing too much (exercise) in a group because it was longer. They (exercises) were the same time but we got more rest periods online".*<br><br>*"I Felt more comfortable at home doing the programme".*

	Improved group interaction with increased use of online platform features.<br><br>Acclimatisation to online environment fostered effective adaptations to communication methods.	*"We did more exercises in the (face to face) group – so I got more breathless and fatigued. I Feel like it's the right amount of exercise online"*<br><br>*"They can send me stronger wristbands (therabands) if its too easy. I progressed up the difficulty – I started with yellow and progressed up the colours – I now have a black"*<br><br>*"The benefits of doing it at home was that we couldn't do it in a group. It was the only option. And it has worked. I would recommend it. It would be better face to face but you've got to go with what's available – a lot of it is outside of our control*

Pulmonary Rehabilitation Class Exercises

Below is a summary of exercises completed during the 6 week online pulmonary rehabilitation course. It is recommended to complete the session in full 3 times a week.

Warm up:

Complete each step of the warm up for 30 seconds (5 minutes in total)

1. Seated toe and heel taps
2. Seated march with arm swings
3. Turn head side to side
4. Roll shoulders forwards and backwards
5. Reaching across your body and twisting your trunk both ways
6. Arms relaxed by your sides, leaning towards the floor both ways
7. Heel digs to each side
8. Standing march – add punching to the front
9. Standing march – add arm bends
10. Start with a small march, make the movement bigger for a count of 5. Repeat this 3 times.

Aerobic Exercises:

Complete each exercise for 3-4 minutes. You should feel a 3-4 on the BORG breathlessness scale whilst completing the exercises. This means you should feel moderately to somewhat severely breathless.

1. Marching on the spot

You can do this standing or seated. Make the movement bigger if you are not feeling breathless.

2. Side step to front punch

Step out to one side with both feet and punch twice to the front. This movement can be done seated. You can lunge further out to the side to make the movement harder.

3. Star Jacks

Move one leg and one arm out to the side simultaneously and then bring them back to the centre. Do the same the other side and repeat. You can do this seated or lunge further out to the side to make it harder.

4. Knee through

Use a chair in front of you for balance if needed. Place one leg behind the other and drive the leg behind through and up towards your chest. Then place the same leg back behind and repeat the movement. Complete half the time on one leg and half the time on the other.

5. Punch with a twist

Place one foot in front of the other for balance. The foot behind is the same side as the arm you will punch with. Punch forwards and twist your torso simultaneously. Bring arm back to neutral position and repeat movement. Complete half the time on one arm and half the time on the other.

Strengthening Exercises:

Aim to complete 10 repetitions of each exercise 3 times. This means you will do a total of 30 repetitions of each exercises with a break after 10 repetitions.

1. Sit to stand

Stand up straight from a chair. Slowly lower yourself back into the chair and repeat. You can use your arms to help push yourself up if you need to.

2. Bicep bends

Start with arms down by your side. Bend arms upwards, keeping elbows tucked into waist at all times. Weights or a resistance band can be used to complete this exercise.

3. Squat to heel raise

Use a chair in front of you for balance if needed. Ensure you keep a straight back and look straight ahead when squatting.

4. Side raise

Use either weights or a resistance band. Keep palms facing down and arms straight as you are bringing them out to the side.

Cool Down:

Hold each stretch for 30 seconds both sides.

1. Start with marching and make the movement smaller over 1 minute.
2. Take a seat and tap your toes and heels for 30 seconds.
3. Neck stretch – put your ear towards your shoulder
4. Roll shoulders forwards and backwards
5. Back stretch – interlink fingers and push arms out in front of you as if you were hugging a tree. Look down.
6. Chest stretch – put both hands in the small of your back and push shoulder back. Look ahead of you.
7. Side stretch – relax both arms by your sides. Lean towards the floor both ways.
8. Leg stretch – place both hands on one bent knee and straighten your other leg. Lean forwards and feel the stretch in the back of your leg.

Risk Assessment Form

TASK	***Risk assessment of home PR – SPACE and ELEARN***	***ASSESSMENT No***		
Source		***Date of Assessment***	31/07/2020	
PREMISES	***Own home***	***PERSON RESPONSIBLE***	Clinician Name	

Consequence

Likelihood	1	2	3	4	5
**1**	1	2	3	4	5
**2**	2	4	6	8	10
**3**	3	6	9	12	15
**4**	4	8	12	16	20
**5**	5	10	15	20	25

Ref No	***Hazard***	***Person(s) exposed to Hazard***	***Risk Identified***	***Pure Risk Rating***			***Control Measures Required***	***In place***		***Residual Risk Rating***		
				**C**	**L**	**R**		**Y**	**N**	**C**	**L**	**R**
1	Room size not sufficient to allow people to move easily during exercise	patient	Risk of injury to patient during exercise	1	1	1	Environmental checklist provided for patient to assess own environment.	Y		1	1	1

							Clinician to view proposed space as part of virtual pre-assessment.					
2	Objects or equipment on the floor in the room constituting a trip hazard.	patient	Risk of falls	1	1	1	Detailed falls assessment for patients with history of falls at pre-assessment.<br><br>Rockwood frailty score to identify those more at risk of falls.<br><br>Environmental checklist.	Y		1	1	1
3	Temperature of room during exercise (excessive heat or cold)	Patients	Risk of dehydration or heat exhaustion in hot conditions.	2	1	2	Temperature should be at least 13ºC minimum (ideally between 18 and 23ºC.<br>If the temperature exceeds 23ºC a decision can be made to not exercise on that particular session using poll on ELearn.<br><br>Open windows for ventilation.<br><br>Patients on Elearn to refer to ‘When not to exercise’ guide.	Y		1	1	1

4	Technology preventing sharing of webcam/ being able to hear class lead	patients	Injury due to unsupervised exercise	3	1	3	Webcams to be activated at all times<br><br>Tech support by staff member if webcam dropping out<br><br>IPad to be provided where necessary	Y		1	1	1
5	Theraband damage causing snapping and potential injury	patients	Muscular injury due to incorrect usage	3	1	3	Instructions on checking theraband for tears prior to every class.<br><br>Multiple bands issued.	Y		1	1	1
6	Confidentiality	Patients, staff	Breaches of confidentiality as family members in the room during the exercise class	2	2	4	Patients and carer only to be in the room for duration of the class. Carer to stay behind screen where possible.	Y		1	2	2
7	Adverse event suffered during exercise	Patients	Acutely unwell due to pre-existing condition or unpredictable acute event	2	6	12	Detailed pre-assessment using guidance from the South West on general principles for remote	Y		1	3	3

			Distressing for the group				pulmonary rehabilitation assessment.<br><br>Pulse oximeter provided for monitoring.<br><br>Rockwood score to guide ratio of staff to patients – no more than three patients with score of 5 or above per class if one moderator.<br><br>Clinician moderating group at all times with phone numbers and addresses available.<br><br>Patient to be moved into breakout room with moderator whilst action taken.					

Consequence X Likelihood = Risk Total (C x L = R)

RED IMMEDIATE ATTENTION REQUIRED Top Priority – must not be allowed to continue ***ALL RED risks MUST be reported to Health & Safety Team***

ORANGE ATTENTION REQUIRED As soon as possible. ***Tel: 08456 598146 (Urgent items/assistance) 07919 401228***

YELLOW RISK REDUCTION REQUIRED As soon as reasonably practicable

GREEN NO IMMEDIATE ACTION REQUIRED Risk is tolerable for the time being, needs reviewing regularly, especially after changes.
